# The efficacy of rimegepant for the acute treatment of vestibular migraine

**DOI:** 10.1038/s41598-026-39902-9

**Published:** 2026-02-16

**Authors:** Heling Chu, Jingwei Pan, Chuyi Huang

**Affiliations:** 1 Department of Gerontology, Shanghai Sixth People’s Hospital Affiliated to Shanghai Jiao Tong University School of Medicine, No. 600, Yi Shan Road, Shanghai, China; 2 Health Management Center, Renji Hospital, School of Medicine, Shanghai Jiaotong University, No. 160 Pujian Road, Shanghai, China

**Keywords:** Vestibular migraine, Rimegepant, CGRP receptor antagonist, Acute treatment, Diseases, Medical research, Neurology, Neuroscience

## Abstract

**Introduction:**

This study aimed to observe the efficacy and safety of rimegepant, a small-molecule calcitonin gene-related peptide (CGRP) receptor antagonist, for the acute treatment of vestibular migraine (VM).

**Methods:**

Patients meeting the diagnostic criteria for VM or probable VM received rimegepant 75 mg for acute attacks. They rated the severity of five prominent VM symptoms (vertigo, unsteadiness or dizziness, nausea or vomiting, photophobia or phonophobia, and headache) on a 0–3 scale. Assessments were recorded at baseline, 30 min, 1 h, 2 h, 4 h, 24 h, and 48 h post-treatment. Efficacy outcomes included the percentage of patients with symptom recovery (from moderate/severe to absent/mild), moderate/severe symptoms, and freedom from symptoms at various time points.

**Results:**

18 VM patients were included. All symptom scores showed a decreasing trend from 30 min after rimegepant treatment. Scores for all symptoms except headache were significantly decreased at 2 h. The percentage of patients with moderate/severe symptoms decreased from 50.0% to 72.2% at baseline to 0% by 24 h for all symptoms. Moreover, freedom from all symptoms except unsteadiness/dizziness was achieved by 24 h. No adverse events were reported.

**Conclusion:**

We first demonstrate rimegepant 75 mg is safe and well-tolerated, which contributes a rapid relief of the prominent symptoms of VM including vertigo, unsteadiness or dizziness, nausea or vomiting, photophobia or phonophobia, and headache. Although required further validation, our findings may provide novel viewpoint on the treatment of VM.

## Introduction

Vestibular migraine (VM) is a neurological disorder characterized by recurrent episodes of vertigo accompanied by migrainous features, including migrainous headache, photophobia and phonophobia, or visual aura lasting for minutes to days. It is considered as an episodic syndrome that may be associated with migraine^1,2^. The pathogenesis of VM is very complicated. Calcitonin gene-related peptide (CGRP), a potent vasodilating neuropeptide in the trigeminal vascular system, is also expressed in the vestibular system and may contribute to VM pathophysiology through the promotion of neuroinflammation^3^. It has been demonstrated that anti-CGRP medications may be an option for the treatment of VM ^4^. Rimegepant, a small-molecule CGRP receptor antagonist, is the only medication effective for both acute and preventive treatment of migraine^5^. However, there has been no research on the efficacy of rimegepant for the treatment of VM. Here, we observed the efficacy and safety of rimegepant for the acute treatment of VM.

## Methods

### Patients

The patients were from Shanghai Sixth People’s Hospital Affiliated to Shanghai Jiao Tong University School of Medicine. The inclusion criteria were as follows: (1) aged > 18 years; (2) fulfilling the 3rd edition of the International Classification of Headache Disorders (ICHD-3)^2^ or International Classification of Vestibular Disorders (ICVD)^1^ criteria for diagnosing VM or probable VM; (3) the attacks lasting at least 2 hours; (4) 2–8 attacks of moderate or severe intensity per month within last 3 months; (5) a stable dose of prophylactic medication for at least three months prior to study enrollment. The exclusion criteria included: (1) history of significant systemic or chronic illness; (2) history of significant neurologic disease or major psychological disorders; (3) potential allergic reactions to rimegepant; (4) women who are pregnant or breastfeeding; (5) refused to be enrolled. This study was approved by the Ethics Committee of Shanghai Sixth People’s Hospital Affiliated to Shanghai Jiao Tong University School of Medicine (No. 2024 − 239). All methods were performed in accordance with Declaration of Helsinki. Written informed consent was obtained from all patients.

## Efficacy evaluation

Patients received paper diaries containing instructions to rate five prominent symptoms of VM, including vertigo, unsteadiness or dizziness, nausea or vomiting, photophobia or phonophobia, and headache on a scale from 0 to 3. The scale was defined as follows: 0 (absent), 1 (mild; bothersome but not limiting), 2 (moderate; limiting activities), and 3 (severe; incapacitating). A visual analog scale (VAS) was also used to evaluate how severely the symptoms of VM disturbed the patients^6^. 0 indicated no disturbance and 10 indicated extreme disturbance. Ratings were to be recorded at the time of taking rimegepant 75 mg (0 min), and subsequently at 30 min, 1 h, 2 h, 4 h, 24 h, and 48 h.

The efficacy outcomes included the percentage of patients with recovery from moderate/severe symptoms to absent/mild, moderate/severe symptoms, and freedom from symptoms after rimegepant treatment at 1 h, 2 h, 24 h, and 48 h. Moreover, the change of scores at all time points after rimegepant treatment was observed. Besides, the adverse events reported by the patients were also recorded.

### Statistical analysis

Statistical analyses were conducted using SPSS 22.0. Data for continuous variables were presented as means±standard deviations (SDs) or medians with interquartile ranges (IQRs) as appropriate, and were analyzed with the two-tailed Student’s *t* test, one-way analysis of variance (ANOVA), Mann–Whitney *U* test, or Kruskal–Wallis *H* test, depending on data distribution and number of groups. The formal two were used as parametric tests when the data conformed to a normal distribution. The non-parametric tests including Mann–Whitney *U* test and Kruskal–Wallis *H* test should be used for comparing the non-normal distribution data. Partial eta-square was used for measuring the effect size of the ANOVA. Categorical variables were expressed as percentages and compared using the Chi-square test or Fisher’s exact test (two-tailed). A *P* value < 0.05 was considered statistically significant. Figures were generated with GraphPad Prism version 9.00. All statistical analyses were performed by statisticians blinded to the groups.

## Results

A total of 18 VM (*n* = 9) and probable VM (*n* = 9) patients (6 males, and 12 females) with the average age of (39.6 ± 14.3) years. Half of them had a history of migraine, and the median course of VM was 5 (IQR: 3-8.5) years (Table 1).

**Table 1:** Demographic and clinical characteristics of the patients.

Characteristics	Patients (*n* = 18)
Age, y, mean (SD)	39.6 (14.3)
Sex, female, n (%)	12 (66.7)
History of migraine, n (%)	9 (50)
Diagnosis	
VM, n (%)	9 (50)
Probable VM, n (%)	9 (50)
Course of VM, y, median (IQR)	5 (3-8.5)
Duration of untreated attacks (hours), n (%)	
2–6	6 (33.3)
6–24	6 (33.3)
> 24	6 (33.3)
Frequency of attacks per month, n (%)	
2–4	11 (61.1)
5–6	3 (16.7)
7–8	4 (22.2)

VM, Vestibular migraine.

**Fig. 1:**
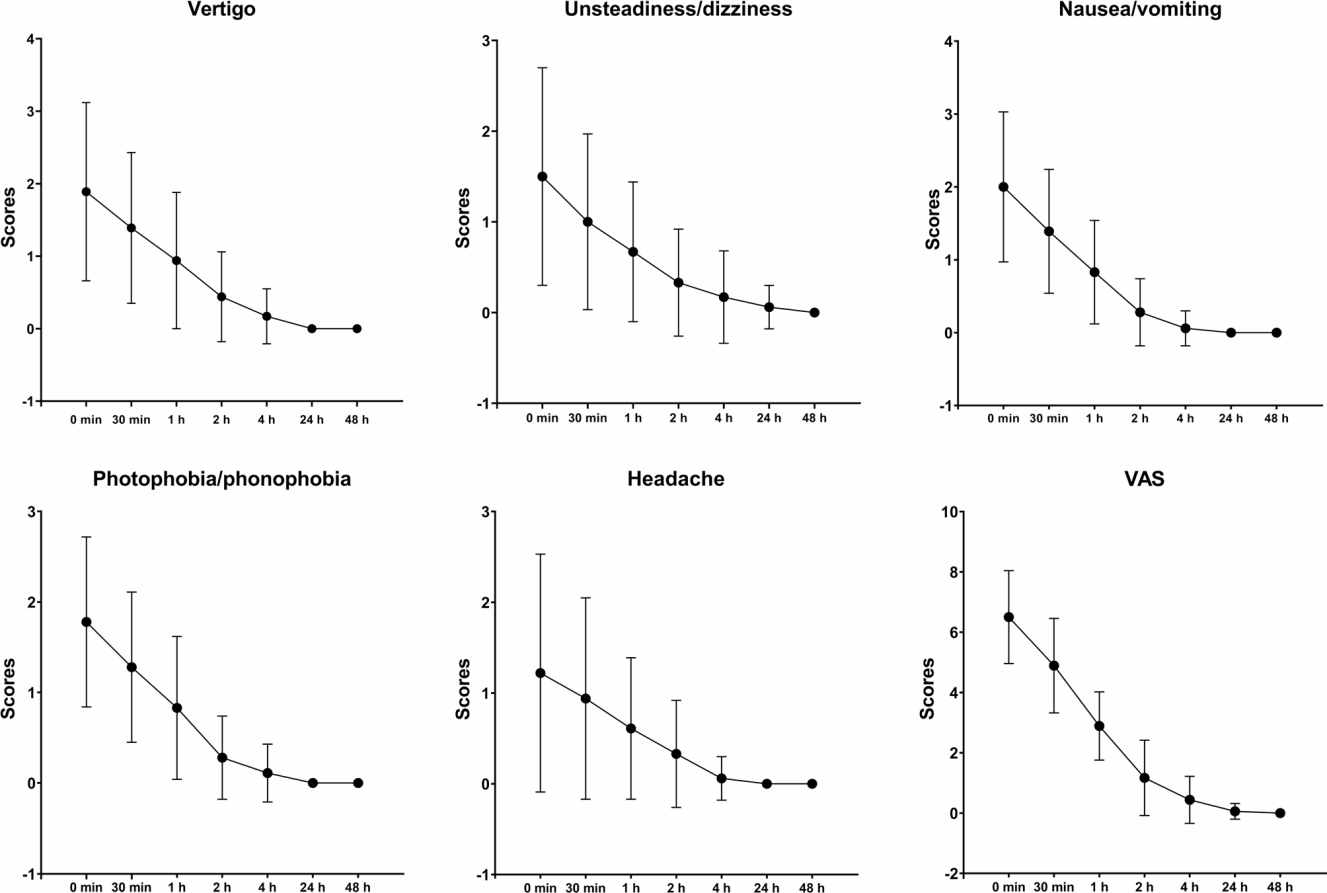
The change of scores assessing the severity of vertigo, unsteadiness or dizziness, nausea or vomiting, photophobia or phonophobia, and headache as well as VAS after treatment of rimegepant at 0 min, 30 min, 1 h, 2 h, 4 h, 24 h, and 48 h.

The scores including vertigo, unsteadiness or dizziness, nausea or vomiting, photophobia or phonophobia, headache all showed a downward trend from 30 min after rimegepant administration. Also, the significantly statistical differences of the scores for all symptoms except headache decreased by rimegepant were observed at 2 h. Rimegepant 75 mg reduced the scores to 0 in each symptom except unsteadiness or dizziness (0.06 ± 0.24) after 24 h, and the latter returned to 0 at 48 h. VAS that assessed the disturbance of VM declined over the time points and returned to 0 at 48 h after rimegepant treatment (Fig. 1).

**Fig. 2:**
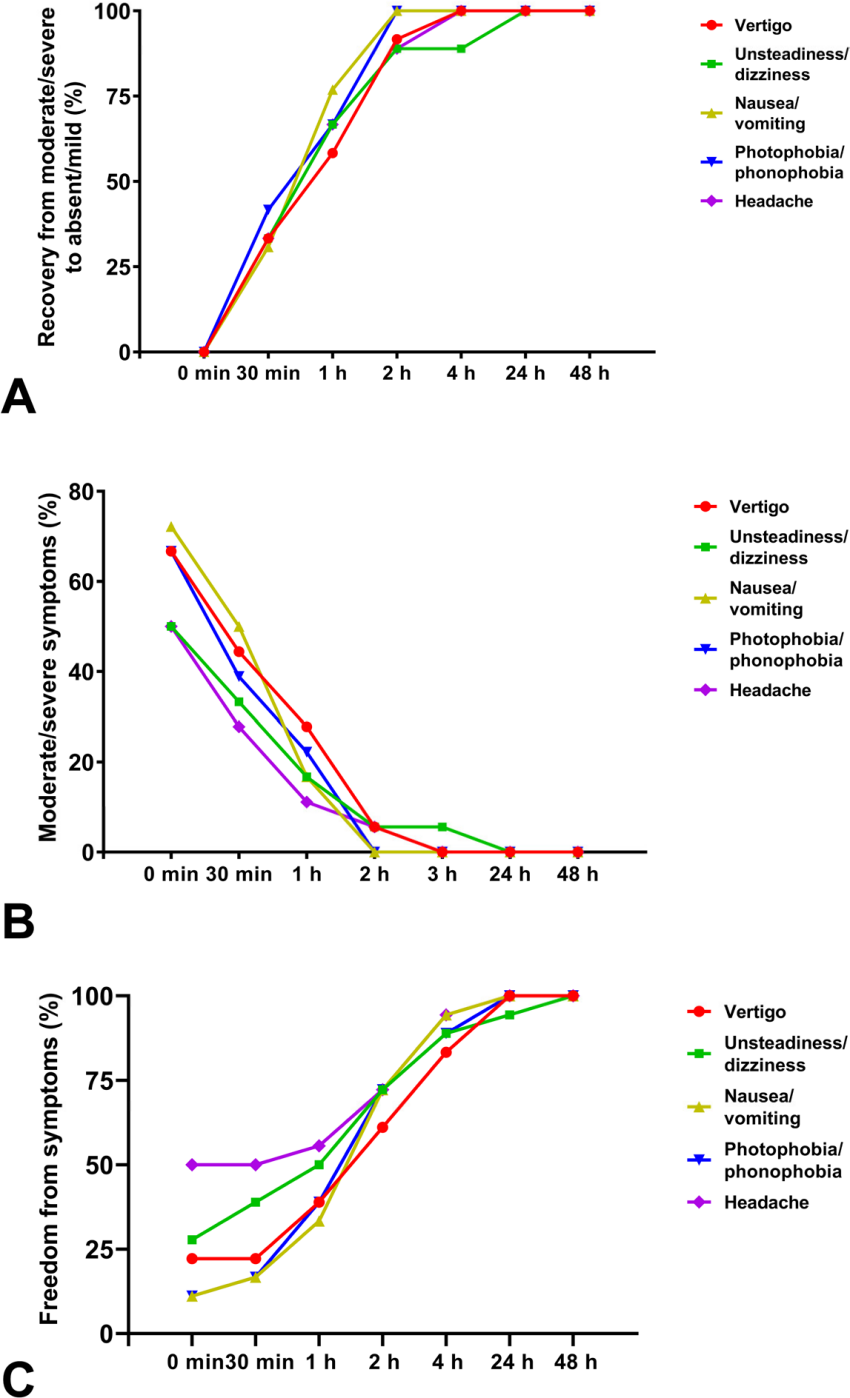
The percentage of the patients with recovery from moderate/severe symptoms to absent/mild (A), moderate/severe symptoms (B), and freedom from symptoms (C) after treatment of rimegepant at 0 min, 30 min, 1 h, 2 h, 4 h, 24 h, and 48 h.

The changing trend of the percentage of patients with recovery from moderate/severe symptoms to absent/mild, moderate/severe symptoms, and freedom from symptoms after rimegepant treatment was similar in all the five prominent symptoms (Fig. 2). As to the efficacy outcomes, all patients recovered from moderate/severe nausea/vomiting and photophobia/phonophobia to absent/mild at 2 h after rimegepant treatment, and all the symptoms reduced to absent/mild at 24 h. Meanwhile, the percentage of patients with moderate/severe symptoms were reduced from 50.0% to 72.2% to 11.1%-27.8% at 1 h, and markedly to 0-5.6% at 2 h after rimegepant treatment, with no moderate/severe symptoms after 24 h. Besides, the percentage of patients with freedom from symptoms increased over the time points after rimegepant treatment, and all the symptoms were relieved at 24 h except 5.6% of unsteadiness/dizziness (Table 2).

No adverse events including allergic reactions, nausea, and liver and kidney function damage were observed.

**Table 2:** The treatment outcomes of rimegepant.

	0 h	1 h	2 h	24 h	48 h
Recovery from moderate/severe to absent/mild symptoms					
Vertigo	0	58.3%	91.7%	100%	100%
Unsteadiness/dizziness	0	66.7%	88.9%	100%	100%
Nausea/vomiting	0	76.9%	100%	100%	100%
Photophobia/phonophobia	0	66.7%	100%	100%	100%
Headache	0	66.7%	88.9%	100%	100%
Moderate/severe symptoms					
Vertigo	66.7%	27.8%	5.6%	0	0
Unsteadiness/dizziness	50.0%	16.7%	5.6%	0	0
Nausea/vomiting	72.2%	16.7%	0	0	0
Photophobia/phonophobia	66.7%	22.2%	0	0	0
Headache	50.0%	11.1%	5.6%	0	0
Freedom from symptoms					
Vertigo	22.2%	38.9%	61.1%	100%	100%
Unsteadiness/dizziness	27.8%	50.0%	72.2%	94.4%	100%
Nausea/vomiting	11.1%	33.3%	72.2%	100%	100%
Photophobia/phonophobia	11.1%	38.9%	72.2%	100%	100%
Headache	50.0%	55.6%	72.2%	100%	100%

## Discussion

The term “vestibular migraine” was first proposed in 1999 ^7^, which gradually replaces the previous used terms such as migrainous vertigo, migraine-associated vertigo, migraine-associated dizziness, and migraine-associated vestibulopathy^2^. Currently, as a commonly underdiagnosed disorder, VM is frequently observed in general practice, headache specialty, and neuro-otology clinical settings, while multiple unknown areas should be further investigated. Research employing standardized methodologies has demonstrated that migraine therapies may also benefit VM. However, recent systematic reviews highlight significant limitations in these studies, including low quality and substantial methodological heterogeneity^8^. Majority of the current studies on VM primarily focuses on preventive treatment, but not all medications effective for migraine are suitable for the prevention of VM. For instance, it has been demonstrated by a randomized clinical trial (RCT) that flunarizine at a dose of 10 mg is an effective therapeutic option for patients with VM, particularly for those experiencing substantial disability from their vestibular symptoms^9^. Besides, another RCT reveals venlafaxine, flunarizine, and valproic acid are effective and safe for VM prophylaxis^10^. However, a double-blind RCT on the prophylactic treatment of VM with metoprolol discontinued after randomizing 130 patients due to poor participant accrual, an issue unrelated to medication safety, and no treatment benefit of metoprolol over placebo was demonstrated^11^. As a result, there is a lack of sufficient evidences to support effective preventive treatment for VM. Especially, very limited evidences support the effective acute treatment for VM. A recent double-blind RCT including 222 participants reveals that the efficacy of rizatriptan in treating acute VM attacks is not observed at 1 h, and its symptomatic benefits at 24 h were marginal, which prevents recommending to use rizatriptan for VM attacks^12^.

CGRP, a 37 amino acid neuropeptide, exhibits pleiotropic physiological effects. The E-group of the efferent vestibular system comprises CGRP-positive neurons located rostrocaudal to the vestibular nuclei, and these neurons project directly to the vestibular end-organs^13^. It has been reported that CGRP plays a significant role in the pathogenesis of VM ^14^. Also, preclinical studies in CGRP knockout mice suggest a potential role for CGRP in the symptoms of VM. These mice demonstrate a reduced vestibulo-ocular reflex and altered otolith function, alterations that may underlie balance problems^15^. As CGRP plays an important role in the pathophysiology of VM, anti-CGRP medications may possess distinctive therapeutic benefits for VM management. These drugs are composed of two families: monoclonal antibodies (e.g. erenumab, fremanezumab, and galcanezumab), and CGRP receptor antagonist (e.g. rimegepant, and ubrogepant). Both retrospective and prospective observational cohort studies with small sample size demonstrates anti-CGRP monoclonal antibodies including erenumab, fremanezumab, and galcanezumab are effective in the preventive treatment of VM ^16,17^. Given that CGRP antagonists are a relatively new treatment for migraine, the evidence supporting their use in VM is still limited. Only one retrospective study shows ubrogepant improves the symptoms of VM in one of two patients^4^. As an oral small-molecule antagonist of the CGRP receptor, rimegepant is considered as an effective and well-tolerable drug for acute treatment of migraine^18–22^. However, there has been no study on the effect of rimegepant on VM. We first used rimegepant for acute treatment to VM in 18 cases. The findings reveal rimegepant rapidly relieves the prominent symptoms, including vertigo, unsteadiness or dizziness, nausea or vomiting, photophobia or phonophobia, and headache without adverse events. As the duration of a VM episode may resolve spontaneously within 24 to 48 h, and our study was lack of a placebo, the comparison of the data in placebo group from other trials may be helpful. It has reported that the percentages of VM patients with reduction in vertigo, unsteadiness or dizziness, nausea or vomiting, photophobia or phonophobia, and headache from moderate/severe to absent/mild at 1 h are 56.8%, 12.4%, 56.2%, 37.1%, and 42.7%, respectively^12^. The data in our work are 58.3%, 66.7%, 76.9%, 66.7%, and 66.7%, respectively, which are numerically higher than those in the placebo group. In addition, it is worth noting that symptom response may be distinct in different sex, migraine history, or other relevant variables which was not investigated in the current study constrained by the sample size.

Our study has some limitations. It is a single-center observation study with small sample size, which may limit the generalizability of the findings. Also, the absence of a control or placebo group may make it difficult to distinguish the rimegepant’s true effect from the natural fluctuation or spontaneous resolution of symptoms as VM patients may have a high placebo response. For example, it has been reported that even a higher percentage of attacks with absent or mild vertigo (primary outcome) in the placebo group is observed than that in the rizatriptan group (56.8% vs. 48.3%)^12^. Moreover, although the sex distribution in our study was consistent with the known higher prevalence of VM in women, no subgroup analyses were performed due to the small sample size. Besides, this study relies solely on subjective symptom scores with the absence of objective vestibular assessments (e.g., videonystagmography, posturography). As a result, further multiple-center randomized double-blind placebo-controlled clinical trials with long time follow-up are warranted for validating the efficacy of rimegepant on both acute and preventive treatment of VM.

In conclusion, our data first indicate rimegepant 75 mg contributes a rapid relief of the prominent symptoms of VM including vertigo, unsteadiness or dizziness, nausea or vomiting, photophobia or phonophobia, and headache, most of which can be relieved within 2 h. Besides, rimegepant is safe and well-tolerated. Although required further validation, our findings may provide novel viewpoint on the treatment of VM.

## Data Availability

Data will be made available on request.

## **Declarations**

### Medical writing

The authors did not receive any medical writing or editorial assistance for this article.

## References

[1] Lempert, T. et al. Vestibular migraine: diagnostic criteria (Update)1. *J. Vestib. Res.* **32**, 1–6 (2022). 34719447 10.3233/VES-201644 PMC9249276

[2] Headache Classification Committee of the International Headache Society (IHS). ;38:1-211.The International Classification of Headache Disorders, 3rd edition. Cephalalgia (2018). 10.1177/0333102417738202 29368949

[3] Furman, J. M., Marcus, D. A. & Balaban, C. D. Vestibular migraine: clinical aspects and pathophysiology. *Lancet Neurol.* **12**, 706–715 (2013). 23769597 10.1016/S1474-4422(13)70107-8

[4] Hoskin, J. L. & Fife, T. D. New Anti-CGRP medications in the treatment of vestibular migraine. *Front. Neurol.* ;**12**. (2022). 10.3389/fneur.2021.799002 PMC8828914 35153979

[5] Kitamura, S. et al. *Efficacy and Safety of Rimegepant for the Preventive Treatment of Migraine in Japan: A double-blind, Randomized Controlled Trial* (The Journal of Head and Face Pain, 2025). 10.1111/head.14995 PMC12455392 40542538

[6] Beh, S. C. & Friedman, D. I. Acute vestibular migraine treatment with noninvasive vagus nerve stimulation. *Neurology* ;**93**. (2019). 10.1212/WNL.0000000000008388 31554650

[7] Dieterich, M. & Brandt, T. Episodic vertigo related to migraine (90 cases): vestibular migraine? *J. Neurol.* **246**, 883–892 (1999). 10552234 10.1007/s004150050478

[8] Calandre, E. P., Bassila, C., Slim, M. & Rico-Villademoros, F. An overview of the current and emerging treatment options for vestibular migraine. *Expert Rev. Neurother.* **24**, 1157–1179 (2024). 39324692 10.1080/14737175.2024.2405739

[9] Lepcha, A., Amalanathan, S., Augustine, A. M., Tyagi, A. K. & Balraj, A. Flunarizine in the prophylaxis of migrainous vertigo: a randomized controlled trial. *Eur. Arch. Otorhinolaryngol.* **271**, 2931–2936 (2013). 24166742 10.1007/s00405-013-2786-4

[10] Liu, F., Ma, T., Che, X., Wang, Q. & Yu, S. The efficacy of Venlafaxine, Flunarizine, and valproic acid in the prophylaxis of vestibular migraine. *Front. Neurol.* ;**8**. (2017). 10.3389/fneur.2017.00524 PMC5641552 29075232

[11] Bayer, O., Adrion, C., Al Tawil, A., Mansmann, U. & Strupp, M. Results and lessons learnt from a randomized controlled trial: prophylactic treatment of vestibular migraine with Metoprolol (PROVEMIG). Trials 2019;20. 10.1186/s13063-019-3903-5 PMC6937687 31888723

[12] Staab, J. P. et al. Rizatriptan vs placebo for attacks of vestibular migraine. *JAMA Neurol.* ;**82**. (2025). 10.1001/jamaneurol.2025.1006 PMC12070282 40354049

[13] Luebke, A. E. et al. Loss of -Calcitonin Gene-Related peptide (CGRP) reduces the efficacy of the Vestibulo-ocular reflex (VOR). *J. Neurosci.* **34**, 10453–10458 (2014). 25080603 10.1523/JNEUROSCI.3336-13.2014 PMC4115147

[14] Raggi, A. et al. Hallmarks of primary headache: part 1 – migraine. *J. Headache Pain* ;**25**. (2024). 10.1186/s10194-024-01889-x PMC11529271 39482575

[15] Russo, A. F. & Hay, D. L. CGRP physiology, pharmacology, and therapeutic targets: migraine and beyond. *Physiol. Rev.* **103**, 1565–1644 (2023). 36454715 10.1152/physrev.00059.2021 PMC9988538

[16] Russo, C. V. et al. Anti-calcitonin gene-related peptide monoclonal antibodies for the treatment of vestibular migraine: A prospective observational cohort study. *Cephalalgia* ;**43**. (2023). 10.1177/03331024231161809 36946234

[17] Kouga, T., Miwa, T., Sunami, K. & Itoh, Y. Effectiveness of Anti-Calcitonin Gene-Related peptide medication in vestibular migraine: A retrospective cohort study in an Asian population. *CNS Drugs*. **38**, 637–648 (2024). 38809343 10.1007/s40263-024-01094-z

[18] Yu, S. et al. Safety and efficacy of Rimegepant orally disintegrating tablet for the acute treatment of migraine in China and South korea: a phase 3, double-blind, randomised, placebo-controlled trial. *Lancet Neurol.* **22**, 476–484 (2023). 37210098 10.1016/S1474-4422(23)00126-6

[19] Lipton, R. B. et al. Rimegepant, an oral calcitonin Gene–Related peptide receptor Antagonist, for migraine. *N. Engl. J. Med.* **381**, 142–149 (2019). 31291516 10.1056/NEJMoa1811090

[20] Yu, S. et al. Rimegepant orally disintegrating tablet 75 mg for acute treatment of migraine in adults from China: a subgroup analysis of a double-blind, randomized, placebo-controlled, phase 3 clinical trial. *J. Headache Pain* ;**25**. (2024). 10.1186/s10194-024-01731-4 PMC11020209 38627638

[21] Iannone, L. F. et al. Effectiveness and tolerability of Rimegepant in the acute treatment of migraine: a real-world, prospective, multicentric study (GAINER study). *J. Headache Pain* ;**26**. (2025). 10.1186/s10194-024-01935-8 PMC11702052 39762740

[22] Wang, J. et al. Patient-reported outcomes of Rimegepant for acute and preventive treatment of migraine in china: a prospective, multicenter, real-world study. *J. Headache Pain* ;**26**. (2025). 10.1186/s10194-025-02197-8 PMC12595842 41204073

